# Cardiac Dilatation

**Published:** 1907-08

**Authors:** 


### CARDIAC DILATATION.

Duckworth states that in dilatation of the heart the main indications for treatment are first to promote the power of the heart's walls, and, secondly, to relieve the venous engorgement which has arisen from their loss of function. The more pressing duty is to unload the venous system. It is seldom that a general venesection from the arm can be justified, unless it be of small amount, not more than six or eight ounces. Direct bleeding is oftener better done by the application of six or eight leeches to the epigastric or hepatic regions. The process of leeching is a source of more relief and comfort than the amount of depletion would indicate. A poultice should be placed over the leech bites, which encourages further depletion and induces a favorable hyperaemia over a large surface. The hepatic congestion should be further relieved by purgation, the best purgative being mercury in some form. It is the best agent for lowering the resistance in the arterioles and capillaries, and thus relieving the labor of the exhausted cardiac walls. The dietary is important. It should be rather dry if it can be borne. Small feeds every three or four hours with solid or semisolid food are best. Much fluid with the meal is objectionable, as it depresses the stomach and the heart. Alcohol is commonly necessary, and is best given as old brandy, not more than two to four ounces in the twenty-four hours being given. Hot water should be given in the morning to promote diuresis; when the heart is once relieved, the urinary flows is more free. Ascites, if urgent, may demand tapping, and extreme oedema of the legs must be dealt with by Southey's trocar. There is often difficulty in securing adequate rest and sleep at night. Recumbency is seldom possible, and the patient may be allowe dto sit in a properly adapted chair with support for the arms and a rest arranged for the head to lean somewhat forward.—Lancet June 15, 1907.

